# Neurocognitive features in male patients with schizophrenia exhibiting serious violence: a case control study

**DOI:** 10.1186/s12991-015-0086-7

**Published:** 2015-12-21

**Authors:** Hiroko Kashiwagi, Noriomi Kuroki, Satoru Ikezawa, Masateru Matsushita, Masanori Ishikawa, Kazuyuki Nakagome, Naotsugu Hirabayashi, Manabu Ikeda

**Affiliations:** Department of Neuropsychiatry, Faculty of Life Sciences, Kumamoto University, Kumamoto, Kumamoto Japan; National Center of Neurology and Psychiatry, Ogawa-Higashi, Kodaira, Tokyo Japan; Tokyo Metropolitan Matsuzawa Hospital, Kamikitazawa, Setagaya-ku, Tokyo, Japan; Department of Psychiatry, Graduate School of Comprehensive Human Sciences, University of Tsukuba, Tsukuba, Ibaraki Japan

**Keywords:** Serious violence, Schizophrenia, Substance abuse, BACS, Executive function, Working memory

## Abstract

**Background:**

The relationship between violence and neurocognitive function in schizophrenia is unclear. We examined the backgrounds and neurocognitive functions of violent and nonviolent patients with schizophrenia to identify factors associated with serious violence.

**Methods:**

Thirty male patients with schizophrenia who were hospitalized after committing serious violent acts were compared with 24 hospitalized male patients with schizophrenia and no history of violence. We evaluated psychiatric symptoms using the Positive and Negative Syndrome Scale (PANSS) and neurocognitive functions using the Brief Assessment of Cognition in Schizophrenia (BACS)-Japanese version.

**Results:**

Repeated-measures analyses of variance on BACS subcomponents z-scores showed that the violent and control groups had different neuropsychological profiles at trend level (p = 0.095). Post hoc analyses of variance indicated that the violent group had significantly better working memory and executive function than the control group. In post hoc ANOVAs also controlling for the effect of the presence of substance abuse on cognitive function, violent or nonviolent group had a significant main effect on executive function but not on working memory.

**Conclusions:**

Patient with violent or non-violent schizophrenia have distinct neuropsychological profiles. These results may help develop improved psychosocial treatments.

## Background

The relationship between cognitive function and violence has been well documented [1, 2]. Non-psychotic subjects who exhibit repetitive antisocial or violent behaviors have deficits in language, memory, and executive function [3]. Impaired executive function was also observed in subjects who demonstrated repeated antisocial or violent behaviors without psychotic disorders, but who had psychiatric diagnoses including antisocial personality disorder, conduct disorder, and psychopathy [4]. Serper et al. suggested that individuals who have executive dysfunction display aggressive behavior because they struggle to cope with their deteriorating mental functioning or daily life stressors [5].

Among patients with schizophrenia, reported associations between neurocognitive performance and violence or aggression have been inconsistent [6, 7]. Some studies indicate that patients with schizophrenia who have a violent behavioral history perform better on tests of executive function than nonviolent patients [3, 8]. Although a nonviolent group was not assessed, Lapierre et al. found that the number of serious violent acts correlated with higher executive function among outpatients with schizophrenia [9]. However, several studies reported no differences in neurocognitive functions (including executive function) between patients with schizophrenia and violent behavior, and those with schizophrenia but no violent behavior [10–14]. In contrast, Barkataki et al. found that compared with inpatients with schizophrenia and no violent history, forensic inpatients with schizophrenia performed worse on executive function tests [15]. Moreover, in two reports, executive dysfunction was found to be a predictor of aggression in patients with schizophrenia, as measured by tools such as the Modified Overt Aggression Scale [5, 16]. These inconsistent findings seem to be the result of differences in study methodologies, differences in the definitions of violent behavior, differences in the severity of the violence, differences in the study settings such as inpatient and outpatient, and differences in the heterogeneity of patient characteristics. Differences between violence that occurs in the community and that during hospitalization may also explain such inconsistencies.

To minimize heterogeneity among patients and their violent behavior, we examined hospitalized subjects with schizophrenia who had a history of serious physically violent behaviors (including murder, attempted murder, and inflicting serious injury), who were receiving treatment under the Medical Treatment and Supervision Act (MTSA; also known as the Act for the Medical Treatment and Supervision of Persons with Mental Disorders who Caused Serious Harm). This relatively new legislative act focuses on offenders with mental disorders in Japan who are considered to be responsive to psychiatric treatment. It aims to promote rehabilitation among individuals who have committed serious harm to others (including homicide, robbery, bodily injury, arson, or a sex crime) while in a state of insanity or diminished responsibility [17]. There were offenses that were committed deliberately due to hallucinations or delusions, but most of these were committed impulsively due to the influence of the hallucinations or delusions. Offenses that were committed deliberately for practical reasons were not subject to the MTSA, even in cases of schizophrenia. Thus, we sampled subjects with relatively uniform psychotic symptoms and violent actions for the current study. We used the Brief Assessment of Cognition in Schizophrenia–Japanese Version (BACS-Japanese version) [18, 19] for neuropsychological testing, which is sensitive to schizophrenia-related cognitive deficits, and has been suggested to correlate with functional outcome.

As we considered the enactment of serious violence, even if under the influence of the hallucinations or the delusions associated with impulsion, to require some degree of executive function (e.g., planning ability), we hypothesized that patients with schizophrenia who exhibit serious violent behavior while experiencing psychotic symptoms would perform no worse on neurocognitive tests (including those of executive function) than nonviolent patients. To explore these hypotheses, we investigated differences among carefully controlled violent and nonviolent patient groups.

## Methods

### Ethical statement

This study was approved by the Ethical Review Board of the National Center of Neurology and Psychiatry. All participants were given a complete description of the study and provided written informed consent.

### Participants

Participants in the violent group were patients hospitalized in the forensic unit of the National Center of Neurology and Psychiatry Hospital in Japan under the MTSA. Each patient met the following criteria: (1) had a diagnosis of schizophrenia according to the Diagnostic and Statistical Manual of Mental Disorders, Fourth Edition, Text Revision (DSM-IV-TR) [20]; (2) was aged between 20 and 65 years; (3) had no organic disorder (e.g., complications such as tumors and other space-occupying lesions in the brain, cerebrovascular disorders, encephalitis, and epilepsy); (4) had committed murder, attempted murder, or caused serious injury to others (the relevant action) in a state of insanity or diminished responsibility; and, (5) was capable of participating in the study, as determined by a multidisciplinary treatment-team evaluation. We excluded women from this study because only two female patients met the study criteria; their relevant actions were attempted murder and serious injury.

Among the 53 males diagnosed with schizophrenia and hospitalized between September 2011 and May 2013, we selected 41 patients as violent group candidates. All selected had committed murder, attempted murder, or caused injury to others. Of the 41 patients, two were excluded because they were in the observation stage; ethical guidelines do not allow research participation during this stage. Six selectees were excluded by the multidisciplinary team to avoid potential patient-staff conflicts, and three refused to participate (not due to psychiatric symptoms). The final sample consisted of 30 violent group participants. Among these participants, 16 had caused injury to others, five had committed murder, and nine had attempted murder. Eight of the violent group participants had a history of substance abuse: four were alcohol abuse, 3 were cannabis, and the other used multiple substances including amphetamines and cannabis. Of the 8 violent cases with substance abuse, 4 had ingested alcohol at the time of the relevant action. One was using synthetic cannabis. The duration of substance abuse may have an effect on cognitive function, however, we could not obtain accurate duration of substance abuse for the subjects of this study. It has been reported that comorbidity with substance abuse contributes to an increase of violent risk among patients with schizophrenia [21]. So, we included the subjects with substance abuse in our analysis reported below. Participants were hospitalized for an average of 330 days (range 25–1061 days). All violent group participants scored either 3 or 4 for strength of relationship between the relevant action and psychosis, according to the Coding Guide for Violent Incidents [22]. The high scores may have resulted from laws requiring the presence of a treatable condition with a strong relationship between the serious crime and psychiatric symptoms for patient hospitalization in a Japanese MTSA ward.

For the comparison group (referred to as the control group), we selected age- and gender-similar individuals from hospitalized patients in a closed general psychiatric ward of the same hospital (average length of stay: 40 days) who met all other violent group criteria, but had no history of physical violence (with scores of ≤1 on the Gunn and Robertson Scale for Violence [23]), or prior hospitalizations for harming others. Among the 35 men hospitalized in the ward, we chose 24 for control group inclusion, who satisfied our selection criteria and matched the ages of those in the violent group. Three of the control-group participants had a history of substance abuse: two were cannabis, and the other was multiple substances, including an amphetamine, cannabis and alcohol. Participants were hospitalized for an average of 18 days (range 1–105 days).

### Procedures

We investigated age, years of education, age of onset, illness duration, history of substance abuse or dependence (excluding nicotine dependence), and violence or criminal records in the patients’ medical records and court files. At the time of BACS-Japanese version testing, we determined the type and dose of psychotropic drugs prescribed. For antipsychotics, we applied the chlorpromazine conversion to estimate daily dosage [24]. For anti-Parkinson agents, we applied the biperiden conversion [25]. For anxiolytic and hypnotic agents, we used the diazepam conversion [24], and summed the values as benzodiazepine equivalents. Symptom severity was assessed by trained psychiatrists using the positive and negative syndrome scale (PANSS) [26, 27].

### Neuropsychological measures

To assess verbal memory, working memory (digit sequencing test), motor speed (token motor test), verbal fluency, attention (symbol coding test), and executive function (Tower of London test), we used the BACS-Japanese version, which was administered by well-trained psychologists.

Z-scores were calculated for each subcomponent score using means and standard deviations based on age and gender data from matched healthy-control Japanese populations [28].

### Data analyses

We conducted statistical analyses with SPSS software for Windows, version 20.0. To compare the violent and control groups in terms of general characteristics, we used the Mann–Whitney U-test. History of substance use disorder was compared between groups using Fisher’s exact test.

Each BACS-Japanese version subcomponent score was normally distributed, except for those measuring working memory, attention, and executive function. To modify each curve to a normal distribution, we performed a logarithmic transformation of these scores.

No general characteristics differed significantly between the violent and control groups. In previous studies, anticholinergic use [29] and the existence of substance abuse [30] were correlated with neurocognitive test performance in patients with schizophrenia. Thus, repeated measures analyses of variance were performed using the six BACS subcomponents z-scores as dependent variables, “group” (violent or control) and “the presence of substance abuse” (11 subjects: yes; 43 subjects: no) as fixed inter-individual (between-subjects) factors, BACS subcomponents as an intra-individual (within-subjects) factor with 6 levels, and anti-Parkinson drug dosage (biperiden equivalents) as a covariate. We used Greenhouse-Geisser values to correct for potential violations in the assumption of sphericity.

Post hoc analysis of variance on each BACS-Japanese version item was performed using anti-Parkinson drug dosage as a covariate whenever necessary. Significance levels were determined at *p* < 0.05.

## Results

General characteristics of the violent and control groups of patients with schizophrenia are shown in Table 1. No main “group” effect (F _[1, 49_] = 0.008, p = 0.929) was found. The BACS subcomponents within-subjects factor was significant (F _[2.976, 145.823_] = 9.941, p < 0.001). The interaction between “group” and “subcomponent” was approaching significant (F _[2.976, 145.823_] = 2.166, p = 0.095).

**Table 1:** General characteristics of the violent and control groups

	Violent	Control	U	p
	(n = 30)	(n = 24)		
	Mean (SD)	Mean (SD)		
Age	44.1 (11.5)	40.3 (10.7)	297	0.273
Years of education	13.1 (2.6)	14.3 (2.8)	279	0.151
Age of onset (years)	25.7 (6.9)	24.4 (7.3)	308	0.360
Duration of illness (years)	18.0 (12.6)	15.9 (11.8)	319	0.470
Mean dosage of antipsychotics				
Chlorpromazine equivalent dosage (mg/day)	845.63 (528.22)	725.77 (391.29)	325	0.541
Mean dosage of anti-Parkinson drugs				
Biperiden equivalent dosage (mg/day)	0.66 (1.35)	0.88 (1.19)	310	0.294
Mean dosage of benzodiazepines				
Diazepam equivalent dosage (mg/day)	8.97 (12.56)	8.97 (8.61)	314	0.413
PANSS positive score	17.03 (5.56)	16.95 (7.13)^a^	284	0.545
negative score	20.20 (7.07)	18.38 (6.51)^a^	272	0.410
general psychotic score	36.80 (9.95)	32.86 (11.51)^a^	232	0.114
Substance abuse (yes:no)	8:22	3:21		0.310

^a^n = 21 (three subjects were not available.)

As this is an exploratory study, we conducted post hoc analyses for “group” that had a nearly significant interaction with the subcomponents as stated above. As determined by post hoc analyses of variance, the violent group performed significantly better than the control group on BACS-Japanese-version measures of working memory and executive function (Table 2). To control the effect of “the presence of substance abuse” on cognitive function, we performed the univariate analyses of variance on the BACS-Japanese version [EX] (−log[3-(Executive function BACS-J z-score)]) and [WO] (−log[6-(Working memory BACS-J z-score)]) using both “group” and “the presence of substance abuse” as factors and anti-Parkinson drug dosage as a covariate. As the results, main “group” effect on executive function was found (F [1, 49] = 6.915, p = 0.011), while main “group” effect on working memory was not found (F [1, 49] = 1.131, p = 0.293).

**Table 2:** Violent and control group BACS-Japanese version score comparisons with post hoc analyses of variance

	Violent group	Control group	F	P	Cohen’s d
	(n = 30)	(n = 24)			
	Mean (SD)	Mean (SD)			
Verbal memory	−1.83 (1.04)	−2.01 (1.50)	0.205	0.653	0.14
Working memory	−0.94 (1.11)	−1.80 (1.67)			
[WO]	−2.06 (0.15)	−2.16 (0.18)	4.162	0.047	0.61
Motor speed	−1.90 (1.13)	−1.92 (1.72)	0.006	0.904	0.01
Verbal fluency	−1.10 (1.13)	−0.93 (1.53)	0.084	0.773	0.13
Attention	−2.44 (1.14)	−3.05 (1.59)			
[AT]	−2.34 (0.11)	−2.40 (0.14)	2.090	0.154	0.48
Executive function	−0.60 (1.88)	−2.98 (3.32)			
[EX]	−1.15 (0.55)	−1.67 (0.48)	12.791	<0.001	1.00

[WO] = −log[6-(Working memory BACS-J z-score)]

[AT] = −log[7-(Attention BACS-J z-score)]

[EX] = −log[3-(Executive function BACS-J z-score)]

## Discussion

Schizophrenia-related violence is diverse and includes cases that vary widely in severity. We carefully selected violent cases among hospitalized subjects treated under specialized legislation and investigated their neurocognitive features using a comprehensive cognitive assessment scale (the BACS-Japanese version). There were no differences from the control group in length of education, age of illness onset, illness duration, drug dosage, or PANSS scores.

We found that, in comparison with the nonviolent group, patients with a history of serious violence tended to have better executive function than the non-violent patients. This result is consistent with previous work by Naudts and Hodgins [1], who reported that patients with schizophrenia and antisocial behavior or violence demonstrated less executive function impairment than those without these behaviors. In contrast, Schug and Raine [2] concluded that patients with schizophrenia and antisocial behavior were distinguishable from those without antisocial behavior by memory impairments, but not executive dysfunction.

Brain imaging studies have suggested that violence in patients with schizophrenia is associated with the amygdala-orbitofrontal system [1], orbitofrontal cortex [31], temporal lobe [32], and hippocampus [33]. Naudts and Hodgins have suggested that, compared with controls, patients with schizophrenia have impairments in the dorsolateral prefrontal cortex (DLPFC) and orbitofrontal cortex, which are involved in control, planning, and goal attainment [1]. Our results suggest that patients with schizophrenia who exhibit antisocial behavior or violence may have less DLPFC impairment than those without such behavior [3] as DLPFC is known for its involvement in working memory [34, 35] and executive function [36, 37]. It is assumed that DLPFC activity is required to some extent to plan and commit violent or antisocial actions. In contrast, higher executive function in schizophrenia is related to both antipsychotic responsiveness, and a good prognosis for social function and employment [16], which are known protective factors against future violence risk [38]. It should be noted that the violent group patients in the present study were those who were considered to be treatment responsive. A more thorough investigation of the relationship between executive function and aggression, violent behavior, social function, and employment prognosis is needed.

This study has a number of limitations. A major limitation is the low sample size with more importance placed on the low number of non-violent patients with substance abuse (only 3), as well as patients abusing different substances. Another major limitation is that the hospitalization durations in the control and violent groups were different. We recruited control subjects from the inpatient population of an ordinary psychiatric ward within the same hospital because our violent subjects were also inpatients. However, although no differences in PANSS scores or antipsychotic dosage were observed between the two groups, we were unable to eliminate the influence of duration of hospitalization and psychosocial therapy, or other conditions on cognitive function. More specifically, the patients in the violent group generally had received a more enriched psychosocial treatment program than the control group, which might have enhanced the cognitive function. Secondly, as already noted, the violent group patients in the present study were considered to be treatment responsive, which may have resulted in a better cognitive function in this group than the nonviolent group patients. In the future, studies assessing patient cognitive function before therapeutic interventions are needed. In addition, the sample size was small because this study was conducted with patients in the MTSA ward of a single hospital and therefore, the findings should be taken as exploratory. Future research should be conducted in a multi-site design.

## Conclusions

In summary, our results suggest that a certain group of patients with violent schizophrenia have characteristic neuropsychological profiles. These findings can be used to support the development of more effective psychosocial treatments and improve our understanding of patients with schizophrenia and a history of serious violent acts.

## Abbreviations

PANSS: positive and negative syndrome scale
BACS: brief assessment of cognition in schizophrenia
MTSA: Medical Treatment and Supervision Act
DSM-IV-TR: diagnostic and statistical manual of mental disorders, fourth edition, text revision
DLPFC: dorsolateral prefrontal cortex
